# Loneliness and social networks of older adults in rural communities: a narrative synthesis systematic review

**DOI:** 10.3389/fpubh.2023.1113864

**Published:** 2023-05-15

**Authors:** Basharat Hussain, Mahrukh Mirza, Rebecca Baines, Lorna Burns, Sebastian Stevens, Sheena Asthana, Arunangsu Chatterjee

**Affiliations:** ^1^Peninsula School of Medicine, Faculty of Health, University of Plymouth, Plymouth, United Kingdom; ^2^University of Plymouth, Plymouth, United Kingdom; ^3^Plymouth Institute of Health and Care Research (PIHR), Plymouth, United Kingdom; ^4^Centre for Health Technology, University of Plymouth, Plymouth, United Kingdom; ^5^Centre for Coastal Communities, University of Plymouth, Plymouth, United Kingdom; ^6^School of Medicine, University of Leeds, Leeds, United Kingdom

**Keywords:** loneliness, social network, social connections, older adults, rural area, rural communities, healthy aging, social isolation

## Abstract

**Introduction:**

Loneliness has significant impacts on the health of older adults. Social networks help to improve psychosocial and quality of life outcomes among older adults. A fifth of older adults live in rural communities, where geographic isolation poses challenges to health. The dynamics of social networks of older adults in rural communities has not been explored.

**Objective:**

To synthesize the literature related to loneliness and social networks of older adults in rural settings.

**Methods:**

A mixed-methods systematic review was conducted through searching six bibliographic databases to retrieve peer-reviewed literature with no time limits. We performed a methodological assessment of each study using a mixed method quality assessment tool. Findings are synthesized thematically.

**Results:**

A total of 50 studies (32 quantitative, 15 qualitative, and 3 mixed methods) were included in this review. Studies were conducted in 17 different countries, the majority from China (*n* = 12), the UK (*n* = 9), and USA (*n* = 9). Findings revealed that social networks of older adults consist of family, friends and neighbors and continue to be developed through participation in various physical and social activities such as walking groups and participation in religious events. Social networks offer informational, functional, psychological and social support to older adults. Through their social networks, older adults feel socially connected and cared for. Successful interventions to improve social networks and address loneliness were those developed in groups or one-on-one with the older adults. The key ingredient to the positive impact of these interventions on reducing social isolation and loneliness was the formation of a social connection and regular engagement with the older adults. However, the review did not find any explicitly reported theory or model on which these interventions are based.

**Conclusion:**

The prevalence of loneliness among older adults in rural areas needs urgent attention. Social network theory and methods can help in developing interventions to address social isolation and loneliness among older adults in rural communities.

## Introduction

Older adults living in rural communities experience challenges with their health and wellbeing. According to European Union, in 2018 there were 101 million older adults (aged 65 years or more) living in the EU and 20 % of these live in predominantly rural areas (1). The challenges for them are worse because of geographic and social isolation (2, 3).

Prevalence of loneliness among different population groups in the society is increasing to the extent that experts consider loneliness as a global public health epidemic (4, 5). A recent systematic review which is based on data from 113 countries found that a sizeable proportion of population experience loneliness globally (6). Surkalim et al. (6) review specifically noted that up to 6.5% of older adults in Northern European countries and upto 24.2% of older adults in Eastern European countries are experiencing loneliness. These findings are consistent with Bandari et al. (7) who noted that loneliness is more prevalence among older people. World Health Organization (WHO) identified that 20–34% older adults are lonely in some countries (8). Loneliness prevalence has exacerbated further during COVID-19 pandemic (9). A study based on 27 countries projected that the number of lonely adults aged ≥50 will increase from 105 million in 1990 to 333 million in 2050 in its sample countries alone (10). Abshire et al. (11) noted that owing to social isolation, ill health, and socioeconomic deprivation, older adults living in rural areas are at higher risk of experiencing loneliness.

Loneliness has adverse physical and mental health effects (6) compounded with social and economic implications. For example, loneliness is linked with feelings of deep sadness, disempowered and worthless (12). Loneliness is also negatively linked with self-esteem and sense of worth, increased vulnerability and risk for anxiety (13) and depressive symptoms (14). These aforementioned ill effects of loneliness further result in decline in cognitive functioning (15) which leads to poor quality of life (16) and may result in early mortality (17, 18). A systematic review conducted over a 25-year period reported loneliness in old age as a predictor of increased suicide ideations (19) which is supported by later studies (20, 21). Loneliness also effects physiological functioning (22) leading to increased risk of malnutrition (23).

It is reported that social networks play a pivotal role in health and wellbeing (24, 25) and perceptions of social isolation and loneliness (2, 25, 26). However, despite the importance of social networks to loneliness, limited research has examined these dynamics specifically in rural communities. Given the unique context of rural areas, both in terms of fostering social relationships and maintaining health and wellbeing, this mixed-method systematic review explored evidence of the impact of social networks on the loneliness of rural older adults. Key terms used in the review are explained in the literature as:

### Loneliness

Loneliness is a perceived unpleasant feeling, which stems from a lack of desired social connections and inconsistency between expected and experienced strength of social relationships resulting in deficit of individual's social network (7). It is based on perceptions, evaluations and responses of one's interpersonal reality, and is expressed through the multifaceted interplay of behaviors, feelings and cognitions (27).

### Social network

Social networks refer to the relationships, such as friendship and collaboration, between individuals and organizations (28). Social networks are composed of socially relevant nodes and ties. Nodes are network members (individuals and organizations) and ties are relations (family, friends, acquaintances, colleagues etc.) among them (29). A social network is a social structure depicting the connections that individuals and organizations form with one another.

It appears that social networks have been defined and studied in various ways, such as using the term social network (30) but also other related terms like social capital (31, 32), social connections (33), social support (34, 35) etc. However, core dimensions of these definitions convey meanings of social connectedness of an individual and receiving social and psychological support from these contacts. The number and types of contacts an individual has would reflect their social network size and structure. Perceived and received support (functional, psychological, and informational) determines the quality of any social network. Within the literature, social networks are also studied in terms of social isolation and loneliness (36, 37). For this review, social networks include all the aforementioned terms and aspects including any form of social connection, social support, social capital or inter-personal relationship of an older adult in rural areas.

### Social connection

Social connection is defined by structural (size of social network) and functional (intensity of social support) aspects of social life, and the quality of social relationships (satisfaction) (38). Social connection is the sense of belongingness, and of feeling connected, close, cared for and valued among family, friends and other social relations. It is an inherent human need and the basis of interpersonal relationships.

### Older adults

The United Nations defines older adults as persons over 60 years of age (39). In the UK, anyone over the age of 65 years is considered an older person (40). By convention, the stage of elderliness is defined according to chronological age. For example, people at age of 65 and above are referred as older adults; 65–74 years old as early older adult and those over 75 years as late older adult (41).

### Rural area

Categorization of areas as rural or urban is usually based on population density. For example, according to the UK Office for National Statistics (ONS) an area is referred as urban if it has a population of more than 10,000, land with a minimum area of 20 hectares (200,000 square meters), while settlements within 200 meters of each other are linked. All remaining areas are categorized as rural (42). The UN Statistical Commission distinguishes urban and rural areas based on population size and density. Rural areas consist of rural grid cells with a density below 300 inhabitants per km^2^. Rural areas have a population < 5,000 and at least 50% population living in rural grid cells (43). According to the US Census Bureau, an urban area has a population of at least 50,000 and a density of 500 people per square mile. All the remaining population, housing settlements and territory is defined as rural (44).

From these definitions it can be concluded that rural area has a population fewer than 5,000 and is less dense, has sparse population, low built in and is located at a distance from urban area.

### Rural communities

Rural communities are small settlements which have a low population density with relative homogeneity and agriculture-related primary activities (45). People in rural communities are usually known to each other and generally have limited and poor access to social services like health, education and employment (46).

## Review question

How do social networks impact the loneliness of older adults in rural communities?

From the identified literature on social networks and the loneliness of older adults in rural areas, the review also aims to address the following sub-questions:

- How older adults in rural areas form their social networks?
- What interventions are there, that address loneliness in older adults in rural communities?
- What social network theories or models have been used to develop social network interventions?

## Methodology

*Ontologically*, this review takes the stance of the existence of multiple perceptions and understandings about loneliness and social networks. In this view, feelings of loneliness and quality of social network are subjective realities and variable in different individual and cultural contexts.

*Epistemologically*, we take an interpretive position. We argue that loneliness and social networks are better understood through interpretive perspective rather than positivistic epistemology which has a narrow focus (47). Moreover, it is noted that healthcare practice is not just based on a positivistic view of the world, rather interpretation-based inquiry (such as narrative synthesis) could inform an holistic view of health, incorporating the feelings and experiences of individuals (48). We used a narrative approach which offers the flexibility of narrating evidence and insights from multiple types of studies (quantitative, qualitative and mixed methods). This is also helpful in building a holistic picture of the review topic, and answer what, how and why questions (49).

### Auxological aspects

We developed a comprehensive search strategy and remained reflexive at all stages of the review. Reflexivity is helpful in identifying any biases and taking steps to address these.

## Methods

This mixed methods review included quantitative and qualitative studies to answer the review questions. We used Daudt et al. (50) framework to select studies for this review. Following the database searches, two team members rigorously applied inclusion and exclusion criteria (BH, MM). For example, we only included studies clearly mentioning their setting (rural), focus (older adults) and studying loneliness and social network together. All included studies were analyzed at the same time and the findings were integrated. The findings of quantitative studies were narratively summarized and integrated with qualitative findings. In this way, the data were transformed according to the data-based convergent synthesis method described by Hong et al. (51) and manualised by the Joanna Briggs (52). The review team has expertise in undertaking various types of reviews and their reviews have been published in high ranked journals (e.g., BMJ Open, Vaccine). We also used team discussions and feedback meetings as a way to address reviewers bias during interpretations of findings from individual study as well as construction of themes.

## Searches

The literature searches were conducted in January 2022 after an iterative development of the search strategy. We consulted an information specialist in development of the search strategy. The strategy comprised blocks of terms representing the concepts in the PICO and comprised subject headings and free text terms for each concept. PICO was structured as:

- Participants (P): older adults age 60 or over
- Phenomenon of Interest (I): social network and loneliness
- Context (CO): in rural community settings.

The following databases were searched: MEDLINE (Ovid), CINAHL (EBSCO), Embase (Ovid), PsycINFO, Scopus, SocINDEX (EBSCO). Full search histories are reported in Appendix A.

## Inclusion and exclusion criteria

Studies were included if they reported primary research into social networks and loneliness of older adults, living in community in a rural setting. Older adults were defined as those aged 60 or over. Social networks included any form of social connection, social support, social capital, or inter-personal relationship. Any studies which described the setting as remote or rural were included even though these definitions differ by country. Studies from any country were included and we did not limit by date.

Studies were excluded if they did not report primary research, if the population was not older adults in a rural setting, and if they did not explore the relationship between social networks and loneliness. Studies were excluded if they were conducted on older adults in care settings (e.g., care home, hospital). Moreover, studies were excluded if they were not in the English language. Conference abstracts, dissertations, policy briefs or documents were excluded.

## Data extraction

All references were exported into EndNote software and duplicates were removed. To reduce the risk of any bias influencing data extraction, as a pilot, two reviewers (BH and MM) independently reviewed the first 500 titles and abstracts. This was followed by a discussion to clarify the screening criteria and agree the decision making.

BH single screened all remaining references independently. Studies meeting the inclusion criteria mentioned above are included. Data were extracted on an Excel sheet for each included study. We extracted the following information from each study: authors, title, year, design, participants, setting, analysis, and findings (see Appendix B).

## Quality assessment

To report the quality of current evidence on the topic we undertook the quality assessment of the included studies. BH and MM assessed quality of studies using 2018 version of mixed methods appraisal tool (MMAT) for quality assessment of the studies (53). This tool was used because our systematic review included studies of mixed methods i.e., qualitative, quantitative and mixed methods. These reviewers independently assessed the quality of studies and any disagreement in the judgements of the two reviewers was addressed through discussion with the rest of the review team (see Appendix B). We decided not to exclude any study based on poor quality. This decision was taken because the evidence base on the topic is sparse. We did not undertake sensitivity analysis owing to mixed methods studies included in the review.

## Strategy for synthesis

According to Hong et al. (51), there are two designs for synthesizing findings in narrative synthesis review: convergent and sequential. We used data-based convergent synthesis design and synthesized qualitative and quantitative evidence together. This helped us present insights on the topic together/coherently, rather in sequential way where qualitative and quantitative findings are presented separately. We used thematic narrative method to synthesize the findings. Firstly, findings from each study were inductively coded. Then, the resultant open codes and examples of excerpts were discussed with the review team before being collapsed into broader themes and synthesized narratively under each review theme (51).

## Findings

**Figure 1:**
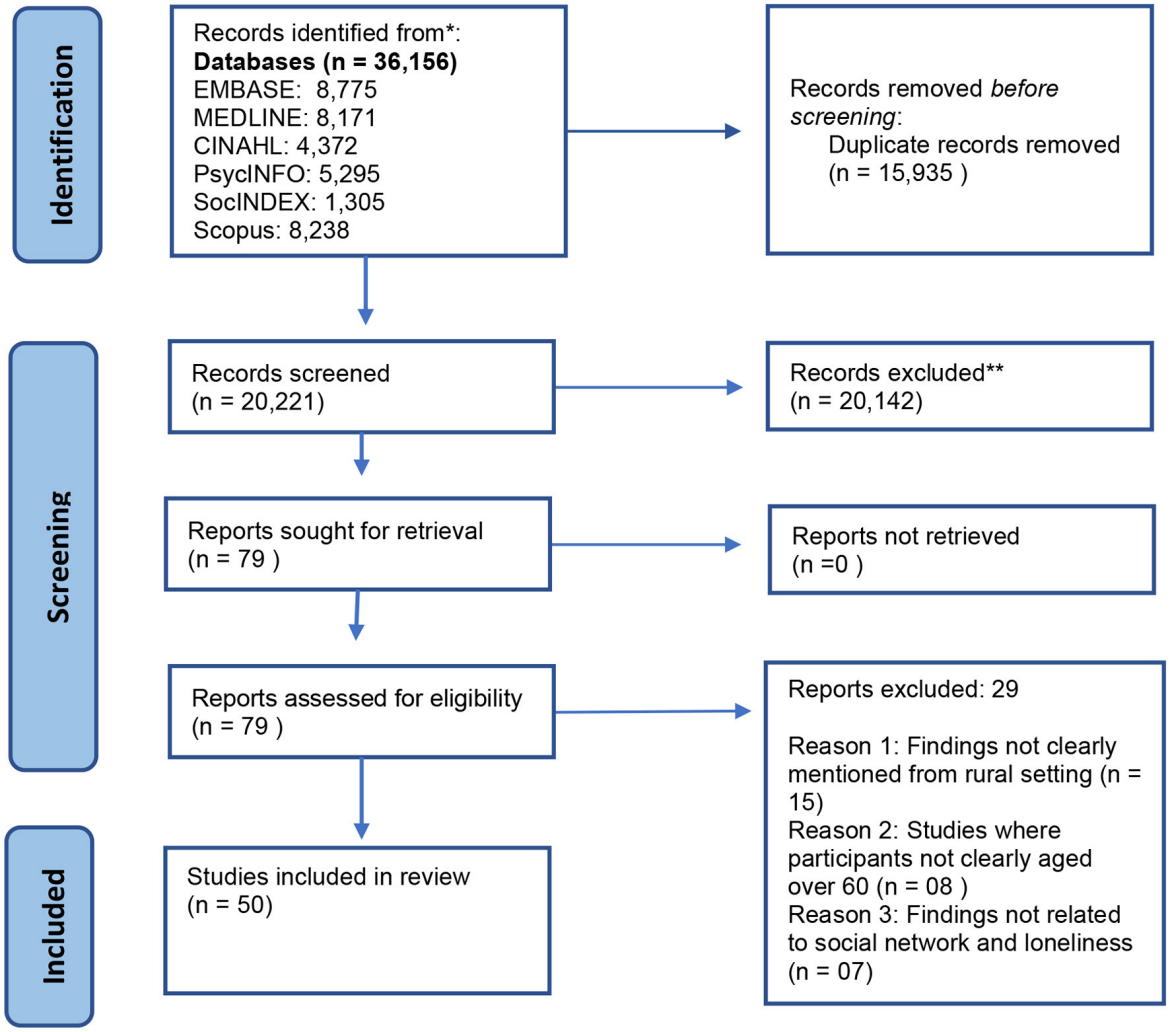
PRISMA flow chart.

The searches identified 36,156 records, which after de-duplication and title and abstract screening, left 79 records for full text retrieval. Following the full text eligibility screening, 29 reports were excluded, leaving 50 studies for inclusion in the synthesis (see Figure 1).

## Overview of studies

**Figure 2:**
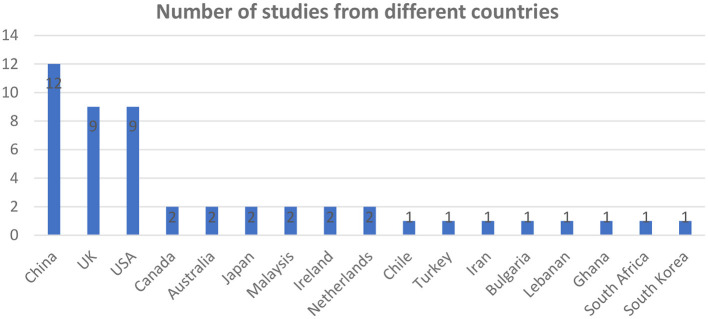
Number of studies from different countries.

In total this review identified 50 studies conducted across 17 countries as summarized in the chart below (Figure 2).

Included studies used quantitative, qualitative, and mixed methods as summarized below:

- Quantitative *n* = 32
  - Descriptive: *n* = 25
  - Cross-sectional analytic study: *n* = 5
  - Quantitative randomized trial: *n* = 1
  - Quantitative non-randomized: *n* = 1
- Qualitative: *n* = 15
- Mixed Methods: *n* = 3

The majority of the studies included were of high quality, *n* = 25 studies were ranked as very good, *n* = 21 ranked as good while only *n* = 5 were of poor quality due to insufficient details about methods.

The review findings are reported into three main themes. These are explained below:

## Theme 1. Development of social networks for older adults in rural areas

This theme is related to the development of social networks among older adults in rural areas. This theme is further divided into three sub-themes. Sub-themes are described below.

### Place and primary groups as key sources of social networks

The review found that place is an important factor in determining social network of an older adult. Compared to urban areas, older adults in rural areas have more structural social support (35). The study measured structural social support for older adults on four items: (i) number of people who share similar interests and are known and have contact with? (ii) number of people known to an older adult to whom they meet or talk to during a week? (iii) How many friends do they have who can visit you in your home and feel “at home”? and (iv) How many people can you speak openly with?

The review found that older adults form primary social networks within their families (54). Spouses and adult children are important part of social networks and also sources of social support for the rural older adults (55, 56). Social interaction of older adults is mainly focused on family members (57), and in some cultures (such as Malays in Malaysia) along with immediate family members, relatives are also part of social network for older adults (56).

As with family contact, persons from rural areas are more likely to have social networks with the neighbors (56, 58). Such networks help to address the issue of loneliness in a better way because living on the same place can ignite a certain sense of belongingness with the place and the people. Neighborhood networks is especially common among older women living alone (59). This indicates that place is an important factor for determining the social network of an older adult. However, a study on social frailty among older adults found that older adults living in community setting in rural areas are lonelier than ones in assisted living facility (60).

### Role of demographic characteristics in contraction of social networks

Age is an important factor in developing or maintaining social network. It is found that older adults become lonelier with the increase in age (61, 62). Kivett (63) found that older rural adults are high risk for the loneliness according to a combination of physical and social losses that they had incurred. In China, depression and age were both significantly associated with loneliness in empty-nest older adults (64). Heenan (57) found that the majority (29) of their participants had experienced reduced social network and some degree of loneliness since becoming 65 years old.

Moreover, the marital status of a person is also an important factor in determining social network. It is found that single older adults have reduced social networks and feel lonelier (65, 66). As people age they loss valuable relations and contacts which make them more vulnerable. Arling (67) noted that widows living in rural areas have limited social network and are more prone to loneliness.

Having a spouse is the most long-term and strongest connection in family related social network in rural areas (55). However, when this connection is lost, widows suffer from loneliness (61, 63). Wenger (61) asserts that the widowed as a group in rural areas are lonelier than others because they do not overcome the trauma quickly and it becomes almost impossible to build new connections as they lose their interest in surroundings for a long period. These findings are confirmed by a more recent study by Jiang et al. (32) which measured Bonding Social Capital (BOC) and Bridging Social Capital (BRC) and evidenced that rural older adults widowed were significantly lower on BOC and BRC, and loneliness of rural participants was significantly associated with both BOC and BRC. Therefore, widows become more prone to loneliness as they get older.

### Role of health and illness status in expansion and maintenance of social networks

For older adults in rural areas, expansion of social networks is achieved through participation in physical and social activities (68). These activities include participating in sports (31), community events and meetings (54). De Koning et al. (33) noted that physical activity can play role in developing new social contacts. In their study, older adult participants in the moderate-to-vigorous physical activity (MVPA) group reported several more sources of social contact compared to those with low physical activity group.

Expansion of social networks is hindered by various factors. These include decline in physical health (56) and disabilities (55, 69). Old age is another barrier that limits social participation as with older age people often experience onset of physical disabilities and chronic illnesses (69). Shergold (70) noted that older age and ill-health are barriers to weekly physical and social activity. These can both hinder the maintenance of an existing social network, and prevent it expanding further. This situation can result in experiencing loneliness, and loneliness itself reduces social participation (71), thus making quality of life for older adults even poorer. Participation in social and physical activities is also hampered by the lack of transportation (68), and fear of crimes (56). Shergold (70) noted that those with car access are up to three times more likely to participate in formal activity (OR 3.228; CI 1.656, 6.293), as compared to those without access to car. Limited access to a car means limited participation in social activities, thus reducing opportunities for expanding one's social network. These factors all contribute to the restriction of social interactions which would otherwise enhance the physical and mental health of older people.

One way to overcome physical disabilities and transport related barriers is the use of technology to get in touch with one's social contacts. Coffee et al. (34) noted that older adults use online socializing as a way to remain connected with their social networks, which was a useful means of communication during the COVID-19 pandemic. Berg et al. (72) support use of technology and consider information and communication technology as enabling connections between older adults and their social networks. Similarly, Willard et al. (73) regarded internet use as important for maintaining social contacts among older adults in rural areas. In using the internet, older adults can increase their online social contacts as well as maintain existing social networks (34, 73). Use of internet can also help the older adults to be exposed to new ideas and information which can help them to interact not only with the people from their age but also from other age groups.

## Theme 2. Social networks and their impacts on loneliness of older adults in rural areas

This theme is related with the strength of social networks and their impact on loneliness of older adults in rural areas. It is further divided into two sub-themes: social impacts and, physio-psychological impacts.

### Social impacts

Social networks play a vital role in establishing feelings of social connectedness among older adults, being able to talk and share thoughts, feel cared for, and supported, and creates a sense of belonging (68). Family is an important part of the social networks of an older adult, offering protection against loneliness (74). Family support also provides older adults with meaningful roles in their family, in turn contributing to their wellbeing, optimism and healthy behaviors (75). Older adults also rely upon family members to provide access to socializing opportunities (57). However, a lack, or absence of family support is significantly associated with loneliness, especially among “empty-nest” older adults in China (64). In addition, a lack of weekly activity with friends and family slightly increases the probability of the loneliness (OR 1.472; CI 0.877, 2.470) (70).

Besides family and friends related social networks, rural participants get support from their neighbors (56). Neighbors offer support in times of need (76), and support from neighbors had a significant positive effect on the older women living alone; as it helped to be able to maintain activities of daily living and brought an improvement in their health status (59). Social network with neighbors works as a coping strategy for loneliness too (34) as reiterated by Sánchez-Moreno et al. (77), where participation in neighborhood and religious groups reduced loneliness.

From the aformentioned studies, it is important to note that social networks offer different kinds of support to older adults. Li and Wang (35) study found that among different types of support, functional social support had a positive effect over loneliness. It is noted that engagement and participation of older adults is important in receiving social support. For example, relationships involving co-engagement were more likely to convey social support (i.e., emotional, instrumental, informational), companionship and social influence (encouragement of healthy behaviors) than relationships that do not involve co-engagement (30). One study (74) indicated that more social support offered protection against loneliness. Relatedly, the influence of living alone was negated by having a good social support system (78).

Moreover, frequent loneliness among older adults is found to be associated with their low participation in organized social activities (63). De Koning et al. (36) argue that volunteering, accompanying others and engaging in sports or exercise were associated with lower social isolation from neighbors, family, and friends. These findings are well-supported by a study conduted in Ireland (79) which noted the intersectionality between social relationships, place, work, health and quality of life among older women.

Similarly, social networks developed through modern technology such as the telephone also have an impact on the loneliness of older adults in rural areas. For example, participants in Hinck (80) study, described their typical day on how they made and received phone calls to deal with loneliness. Participants took an active role in communicating with others by telephoning (80), and a relatively new study by Evans et al. (56) suggested telephone use strengthens social support.

### Physio-psychological impacts

A decline in social relationships and social support led to a higher level of depressive symptoms and low level in quality-of-life scores (69, 81). It is also found that social support partially act as a mediator for depression (82), and those with self-reported depression had significantly higher levels of loneliness (83).

The perceived social support was positively correlated with psychological resilience (84). The study by Hinck (80) supports Yue et al. (84) reporting subjective life experiences of participants highlighting how positive memories of those past experiences strengthened their psychological resilience and have helped them in coping with loneliness (80). Along with memories, there are some other factors like self-confidence, self-worth, and perceived respect which may have an effect on an individual's psychological resilience (85). Van der Geest (85) propose that damage to one's notion of perceived respect toward themselves constitutes an experience of loneliness in older adults. With aging, people tend to become more sensitive about being listened to or given opportunity to impart wisdom and advice to younger people. However, in Van der Geest (85) study, the older adults reported feeling unheard, and a lack of respect. This affected their psychological state and the relationship between the social support they receive and their feelings of loneliness.

As people age, they have reduced social networks and consequently they feel loneliness (61, 63). Lack of social networks results in social isolation and detachment from society (54). A detached older person feels no attachment or lacks a sense of belongingness with the larger group; which results into lower emotional support and is correlated with depression and higher loneliness scores among older adults (86). Higher loneliness in turn results in lower engagement in activities particularly self-care, leisure, and socializing (83).

Coffee et al. (34) viewed increasing social networks in the community as a strategy to cope with loneliness of older adults in rural areas. From their study, De Koning et al. (33) posit that being socially active helps in staying mentally well. There is evidence that any opportunity for social interaction would help reduce loneliness among older adults (87).

Moreover, having poor social network and feeling socially isolated is linked with malnutrition among older adults (37). In another interesting study, it is found that poor social network and feelings of loneliness leads to admission in nursing homes for older adults in rural areas (88).

## Theme 3. Interventions to address loneliness among older adults in rural areas

The review identified a variety of interventions to address loneliness among older people, based on their differing purpose, underlying mechanisms for action and expected outcomes. The purpose of the interventions included: increasing physical activity and social connectedness of older adults (68), enhancing social participation (89, 90), building social capital and friendship (31), mediating loneliness and perceived stress among older people (91), enhancing connectedness, informal care giving and local participation (73), improving general mental health, nutritional status, satisfaction with life, and social capital (92) tackling social isolation and loneliness (58, 93–95).

This theme is further divided in three sub-themes. The description of sub-themes is given below.

### Social and educational interventions

These interventions offered range of social and educational activities to the older adults. For example, in the UK, a village services project offered six community-based services and activities that provided help to meet the needs of older rural residents in three regions of England (95). The services include: (i). Warden service- Community wardens giving emotional and practical support to housebound/lonely, bereaved and convalescing older adults, (ii). Lunch club—A parish center lunch club, part of a county-wide initiative to grow community self-help networks, (iii). Welfare rights—A dedicated worker helping older residents of rural villages in former mining communities access benefit entitlements, (iv). Befriending—Two linked befriending services providing a regular social visit for lonely, isolated clients in their own homes, (v). Information and advice Service—offering information and advice on benefits and services to older adults in dispersed rural areas, including a dedicated worker to visit older adults in their homes to help clients access benefit entitlements, (vi) Lunch club/mobile care service—Transport to a regular social event/meal combined with the delivery of mobile hand, foot and hair care to older people living in remote rural settings.

Likewise, a befriending intervention (58) and the Young at Heart group (57) are both Ireland-based interventions. The befriending intervention administered a weekly home visiting service led by volunteer befrienders intending to facilitate social interaction as well as community-based social groups, activities and outings among the befrienders and befriendees (58). The Young at Heart group is a community-based initiative started in 2008 in Northern Ireland (57). The group aimed to identify needs of local older adults in a participatory way and meet the identified needs. The group identified that older adults need opportunities to socialize, develop skills and obtain information. The group organized various activities for older adults including tea-dances, whist-drives, health information, social security information, local history talks and events, storytelling, outings, information technology & transport, and opportunities for networking with other groups. Similarly, another intervention from China known as Community Canteen offered the opportunity for rural older adults to eat lunch and dinner together each day (92).

Furthermore, online assistance and interventions have also been tested. These interventions are beneficial in resolving major hindrance on the way of generating social support (68), and other physical disabilities (69). In Netherlands, an online community care platform called as “Grubbenvorst-Online” offered apps to older adults living in rural area of Grubbenvorst (73). These apps offered a matching tool for informal care where users could exchange informal help, a local calendar on local events and activities, and social services in which users could find information about available care services and organizations.

Apart from massive interventions, for two different interventions driven-game shooting (31) and Community Canteen Service (92) noted statistically significant, positive impact on mental health and wellbeing, and social capital of the participants where social capital of the Canteen Group was better than the Non-Canteen Group along with improvements in life satisfaction. Latham-Green et al. (31) claimed that the intervention created social impact via social capital creation and identity reinforcement.

The Educational Program for Social Participation in Iran delivered 5 weeks long weekly 60–80 min educational sessions to older adults for encouraging them to increase their social participation and activities in the society (89). This program noted significant improvement in loneliness feeling scores of the intervention group from 62.24 ± 0.7.53 to 28.86 ± 6.88 (*P* < 0.001), however, the control group experienced no significant changes in feelings of loneliness scores (89).

### Psychological interventions

A variety of psychological interventions to tackle loneliness among older adults have been carried out. In China, Chinese Traditional Festival Activities based Group Reminiscence Therapy (CTFA-GRT) was a psychological health promotion program for older adults (91). This program integrated the situational memories of group reminiscence therapy into traditional Chinese festival activities, making it easier to help older adults living alone find a theme of common reminiscence. These are a series of days based on international celebrations and religious events such as International Women's Day (Enhance positive experience), Mother's Day (Recall maternal love), Chinese Valentine's Day (Let go and feel loved).

A social prescribing pilot project for older adults in rural area of South Korea during COVID-19 pandemic offered weekly 10 weeks interventions including music storytelling, a self-help group, gardening, and COVID-19 prevention in 2020 (90). These interventions contributed positively to psychological wellbeing of the older adults by increasing the social connections of older adults and reducing their loneliness.

Moreover, many of these activities were group-based and implemented in different societies across the globe. For example, Active Aging Program—a community health promotion program in British Columbia (Canada) offered walking groups, Garden Club activities, swim programs, Tai Chi, peer support, bee garden, meals, carpet bowling, language education, tea socials, outings by bus, community meals and mobility clinics (68). These activities were contextualized to meet the needs of local populations across British Columbia.

Three interventions: Social Prescribing Intervention, Education Program for Social Participation, and Active Aging Program all found that social participation scores increased for the sample that received these interventions. And it is noteworthy that these studies were conducted in different countries yet achieved the same outcomes such that, social participation scores increased as a result of these interventions (68, 89, 90). The social prescribing intervention resulted in an increase in the social participation attitude score. Although the average score of self-efficacy increased, it was not statistically significant (90). Pearce and Lillyman (94) found that self-worth levels increased with social connections. The Active Aging Program found that older adults through participation in different activities developed social connections, had opportunity to talk and share their thoughts, felt cared for and created a sense of belongingness (68).

Along with improved social participation scores, a positive effect on loneliness was also noted as a result of these interventions. The Social prescribing intervention provided positive outcomes in terms of loneliness (90) such that, loneliness was reduced significantly, while self-esteem increased significantly (94) and depression was also noted to get reduced considerably. The Chinese Traditional Festival Activities and based on Group Reminiscence Therapy (CTFA-GRT) also noted positive outcomes for the participants for the perceived stress and loneliness of rural older adults living alone in the intervention group (91). Combined with a simple effects test, the PSS and UCLA-LS of those in the intervention groups significantly decreased at 8 months after the baseline (91). In addition, the sustainable effect of this program lasted 3 months after the intervention (91). Therefore, it can be said that these interventions not only reduced loneliness in its participants, but reduced loneliness sustained for next 3 months outside the intervention (91).

### Sports and art based interventions

Many interventions used different activities, hobbies, and sports for older adults to connect with each other during these social engagements (31, 58, 90). For example, Bantry-White et al. (58) promoted similarities between group members who shared the rural areas and called it a befriending intervention. By sharing a common history that they co-constructed by using local old photographs and poetry they created a sense of attachment among them that let befrienders and befriended developed friendships and connections through activities such as knitting, baking, bingo, dancing, and gardening. Similarly, Driven Game Shooting in England offered regular involvement of older adults in a rural and countryside sport (31).

Art is a stated means to promote expression and discourage feelings of loneliness (93, 94). MacLeod et al. (93) claimed intervening through arts is a quality effort. In the UK context, an art program implemented creative arts projects named “Extend,” “My Story,” “The Rural Pub Arts Hub” to reduce loneliness among older adults in rural part of Hertford (94). Extend—offered dance sessions involving gentle chair-based exercises to music and are aimed to teach techniques to prevent falls and improve mobility, balance, and independence. My Story was a reminiscence project that captures the stories of older adults on film. The films provided insights into the lives of those involved and enable deeper relationships to develop between the day care staff, families and older adults as conversations arise from the previously unknown stories and interests. The Rural Pub Arts Hub project held accessible beginners' art classes for the over-sixties in rural pubs. The sessions ran over 10 weeks and were led by local artists who introduce painting with watercolor, drawing with watercolor pencils and water-soluble fiber tip pens. Each class worked toward creating a mural, as well as working on collages, stitched imagery and mark-making, and each participant created their own artist's book.

A second art based intervention program from Canada called “Seniors Connecting with Seniors through Expressive Arts Making”—consisted of in-home volunteer-based expressive arts (93). In this intervention, older adult volunteers were matched one-to-one with eight socially isolated older adults to conduct in-home, individual, intermodal art making activities in their dyads over 10 sessions. Among others, these volunteers were retired artists, teachers, and nurses.

## Discussion

Our review finds that family, friends, and neighbors are important sources for developing social network for older adults in rural areas. These social networks are developed through living in a particular area as well as through participation in various physical and social activities. Older adults receive informational, functional, psychological, and social support from their social network. Having good social network generates feelings of being socially connected and valued among the older adults. Our review also found that loneliness of older adults in rural areas can be reduced through group as well as one to one interventions. These findings are similar to other systematic reviews (96–98). Characteristics of effective interventions included their adaptability to the local and individual context, a community development approach, and productive engagement with older adults (99). However, it is also important to remain cautious as there is no one-size-fits-all approach to address loneliness among older adults; therefore, it is important to tailor interventions in a way that suit the needs of individual older adults, specific population groups of older adults and the extent of loneliness being experienced (100). Although our review noted that video calls and online forums helped to reduce loneliness, a robust literature review suggested that there is uncertainty in the evidence to support that video calls are helpful in addressing loneliness among older adults (101).

Caution is also needed in making assumptions that older adults with weak social ties utilize more health services (including physician visits and community- or home-based services) than required by their actual needs (102).

Our review identified that causes of loneliness among older adults in rural areas are varied (103). Marital status, loss of spouse, health issues, limited social network and low level of physical and social activity can all cause loneliness among older adults in rural areas. These risk factors for loneliness are similar to a recent review undertaken to identify longitudinal risk factors for loneliness among older adults in the UK (104) and Europe broadly (105). In our review, we also identify that age is an important social variable associated with loneliness. We identified that loneliness can increase with age suggesting that older adults are more likely to feel lonely. These findings are similar to a 24 years longitudinal study conducted in the UK (106).

A number of theories have been proposed to explain the cause of loneliness (e.g., the existential, the cognitive, the psychodynamic and the interactionist) (107). However, to our knowledge none of the studies have used social network theory to explain causes of loneliness. In our view social network theory can be very helpful in explaining loneliness among older adults living in rural areas (108).

In addressing loneliness, our review suggests that the interventions that develop and/or strengthen existing social networks of the older adults are helpful (99). The exact mechanism through which social networks influence health (109) or specifically reduce loneliness is still unknown (31). However, the underlying mechanism relating to positive influence of social network on loneliness could be due to mainly two reasons:

Firstly, a social network offers opportunity for the older adults to participate in physical and social activities and remain engaged (110). This shifts the cognitive thinking process of older adults toward social activities, rather than their loneliness. The mind of older adults remains preoccupied in social activities and this prevents developing feelings of loneliness (110, 111). It is considered that regular engagement in physical activity, remaining in touch with family contacts and social participation decreases physiological distress among older adults (112).

Secondly, having social network gives feeling of social connectedness to older adults (113). It is identified that these feelings of connectedness with other and to a community or neighborhood contributes to wellbeing of older adults (114). Older adults trust their social surroundings and feel less anxious (115). This develops an environment of reciprocity and feelings of physical, psychological, and social security for the older adults. An older person can get information, emotional, and psychosocial support, and hope that in time of need, support from social connections would be available. Feeling secure and being in a reciprocal relationship reduces social isolation and loneliness (116). In parallel to a recent rigorous study on social network and mental health (117), our review also underlines the need to address the subjective factors of social isolation (such as feelings of loneliness) and objective factors of social isolation (such as social network size) in a complementary way to achieve the goal of improving mental health of the older adults.

Our review identified important evidence gaps in the topic of our interest. A range of terms were used in the reviewed literature to convey the meanings of social network (108). These terminologies include social connections, social support, social capital etc. The use of an agreed upon definition of social network in healthcare research would help in enhancing credibility of the evidence.

Methodologically, we observed that there is gap in the use of social network specific methods and techniques in healthcare research (118). If we intend to understand the concept of social networks in healthcare, Social network specific methods and techniques must be used in undertaking studies (119).

Loneliness among older adults can result in poor health and wellbeing including cognitive impairments such as dementia (120). Therefore, there is an urgent need to generate evidence regarding the mechanisms through which social networks reduce loneliness of older adults in rural areas (119). Studies on this topic need to be robust and conducted in different cultural contexts (121). Studies related to social network and loneliness of older adults from minority population groups such as minority ethnic background living in rural areas of developed world are also needed (122).

## Strengths and limitations

Our review included studies with different study designs including, quantitative qualitative and mixed methods, however most studies used different tools to assess social support, loneliness and sample sizes varied across different studies this made synthesize of the data challenge. We included few interventional studies to address loneliness and showed positive benefits of the intervention in reducing loneliness and promoting social participation, however, the sample sizes were very low in these studies. The majority of the studies included were of high and, very good.

Most of the studies were conducted in China, USA and UK with only two studies from other continents such as Africa. Therefore, the evidence from this review may not be generalisable to all countries.

## Author contributions

All authors listed have made a substantial, direct, and intellectual contribution to the work and approved it for publication.
